# Elucidating the semantics-topology trade-off for knowledge inference-based pharmacological discovery

**DOI:** 10.1186/s13326-024-00308-z

**Published:** 2024-05-01

**Authors:** Daniel N. Sosa, Georgiana Neculae, Julien Fauqueur, Russ B. Altman

**Affiliations:** 1 Stanford University, Department of Biomedical Data Science, Stanford, CA USA; 2 BenevolentAI, London, UK; 3 Stanford University, Department of Bioengineering, Stanford, CA USA; 4 Stanford University, Department of Genetics, Stanford, CA USA

**Keywords:** Knowledge graphs, Knowledge inference, Semantics, Network topology, Drug discovery

## Abstract

Leveraging AI for synthesizing the deluge of biomedical knowledge has great potential for pharmacological discovery with applications including developing new therapeutics for untreated diseases and repurposing drugs as emergent pandemic treatments. Creating knowledge graph representations of interacting drugs, diseases, genes, and proteins enables discovery via embedding-based ML approaches and link prediction. Previously, it has been shown that these predictive methods are susceptible to biases from network structure, namely that they are driven not by discovering nuanced biological understanding of mechanisms, but based on high-degree hub nodes. In this work, we study the confounding effect of network topology on biological relation semantics by creating an experimental pipeline of knowledge graph semantic and topological perturbations. We show that the drop in drug repurposing performance from ablating meaningful semantics increases by 21% and 38% when mitigating topological bias in two networks. We demonstrate that new methods for representing knowledge and inferring new knowledge must be developed for making use of biomedical semantics for pharmacological innovation, and we suggest fruitful avenues for their development.

**Supplementary Information:**

The online version contains supplementary material available at 10.1186/s13326-024-00308-z.

## Introduction

Artificial intelligence holds great promise for discovery and innovation in pharmacology from identifying new drug targets to predicting new applications for old drugs, or drug repurposing. Underpinning these innovations is an understanding of normal human biology and of the pharmacodynamics–how a drug affects the body–and pharmacokinetics–how the body processes a drug–of drug response. Critically important are interactions between several biological entities, namely drugs, diseases, proteins, and genes.

Knowledge of these interactions can be represented well as a knowledge graph (KG), a simple and flexible network data structure. KGs facilitate computation and are amenable to network methods for addressing complex questions like how to repurpose a drug for a novel condition by framing the task as predicting new links in the graph [1, 2].

Gold-standard databases that would comprise pharmacological interactions between drugs, diseases, genes, and proteins are manually curated [3]. While these benefit from human quality assurance, they suffer from limited coverage due to the limited capacity of manual curators and the rapid proliferation of biomedical literature.

Advances in natural language processing present an opportunity to increase the coverage and scope of these KGs by automatically extracting relations between relevant entities from scientific text. Already multiple such global knowledge graphs have been created from biomedical literature at the scale of PubMed [4, 5].

Equipped with large-scale KGs, machine learning methods can be leveraged for pharmacological discovery. The primary class of methods, known as KG embedding methods, learns numerical representations of entities and relations in a KG to automatically infer new links implied by existing knowledge [6]. These inference methods have been applied to tasks including KG completion, question answering, and logic prediction generation [6, 7]. In drug discovery, these methods are poised to predict drug repurposing opportunities [2], disease-gene associations [8, 9] and drug-target interactions [10].

Extracting knowledge directly from text has the benefit of capturing the rich semantics of the relationship between entities, which represents biological nuance. However, systems for relation extraction are imperfect and suffer from noise, missed key syntactic clues such as negation, and challenges in discerning relevant knowledge to extract [11–14]. Further complicating automated knowledge-based systems for pharmacological discovery, it has been shown that knowledge embedding methods, the primary class of knowledge inference methods, suffer from network topology-based biases, where the presence of highly connected, or “hubby”, nodes inflates evaluation metrics of inference quality [15]. These features of global KGs must be carefully considered in understanding the quality and caveats of discovery driven by large knowledge systems.

In this work we provide the first analysis of the interrelation between relation semantics and topology. We aimed to assess the capacity of knowledge embedding models to leverage knowledge graph semantics for pharmacological inference. We demonstrate that in the presence of network topologies with highly variable node degrees and wherein a small subset of nodes are highly connected hubs, the benefit of nuanced semantics is diluted, suggesting that new methods must be devised that make use of this important biological information with equal potency as the network structure itself.

## Related work

Recent work in knowledge inference has shown that computational successes are very sensitive to experimental conditions. In a comparison of commonly used embedding methods, Berrendorf et al demonstrated that results were sensitive to the chosen model architecture, the training approach, the loss function, and certain data assumptions [16]. Another study showed that these factors and others including model parameter initialization and different splits of the datasets have great impacts on results for applications using drug discovery-oriented knowledge graphs, demonstrating the pertinence of these considerations in the biomedical domain [17].

In parallel, the topic of knowledge graph quality assessment is well studied in the field of semantic technology. Zaveri et al. provide an overview of quality assessment for linked data, describing many commonly used metrics such as accuracy, timeliness, completeness, relevancy, consistency, availability, and verifiability [18, 19]. The notion of consistency is particularly pertinent, which concerns the absence of logical contradictions in the knowledge graph [20]. SemMedDB, for instance, is a global literature-scale knowledge graph that has been demonstrated to have over 500,000 inconsistent triples [21]. It has even been shown that when quality checks are evaluated for large benchmarking knowledge graphs, consistency, completeness, and accuracy can vary widely [22]. Lowering the quality of knowledge in KGs via increasing the levels of incompleteness or noise have been shown to lead to large degradations in performance for KG completion [23].

Work is emerging linking network topology as a confounding factor for knowledge inference methods. Zietz et al showed that a competitive baseline for inferring link prediction, which they call an “edge prior”, can be constructed using node degree alone [24]. The authors show that using edge priors for link prediction performs well on biomedical prediction tasks including drug-disease prediction, disease-gene association, and drug-target binding. Another work by Bonner et al reinforces this finding by showing that knowledge graph embedding methods for biomedical link prediction also favor high-degree nodes yielding performance metrics that appear inflated [15]. This observation was consistent across a variety of inference tasks and embedding methods.

## Materials and methods

**Fig. 1:**
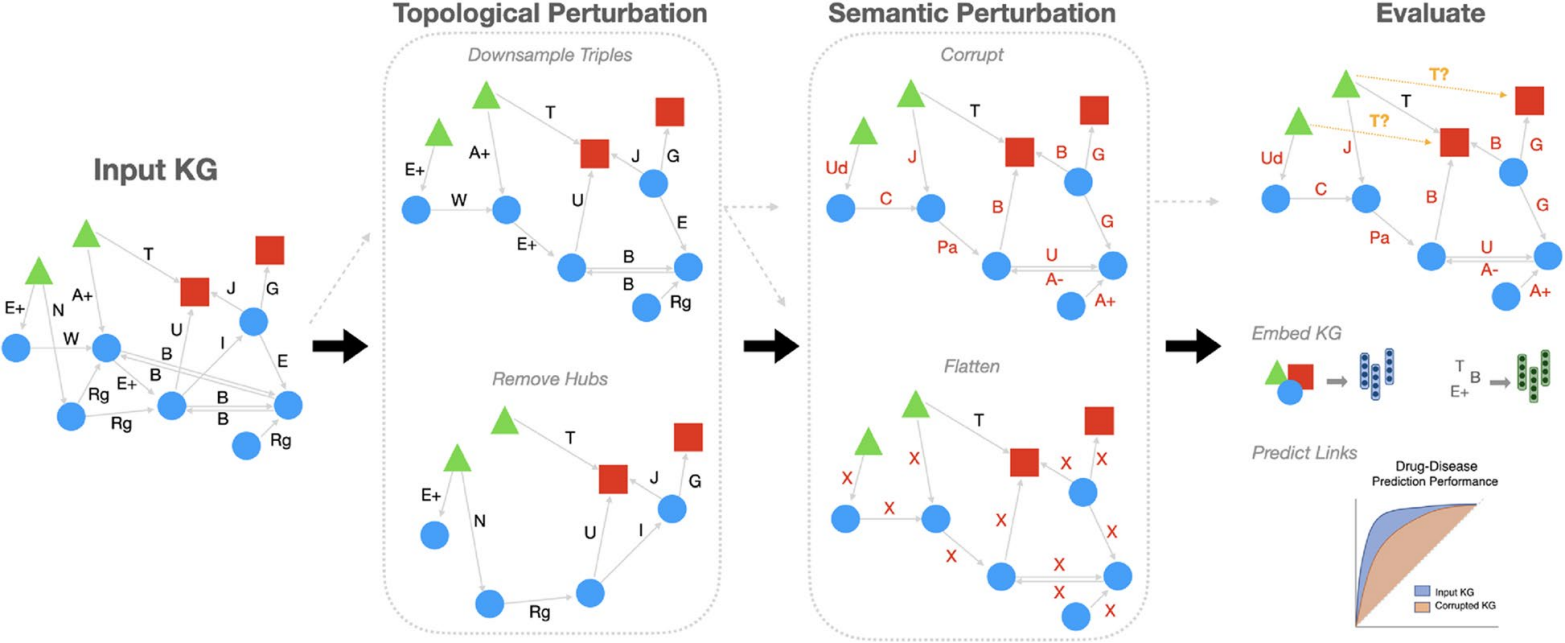
Overview of the KG processing and evaluation pipeline. Input KGs are first pre-processed by altering their topology via degree-based downsampling or hub removal. The semantics of KG relations are experimentally perturbed via corruption or flattening down to a single edge type for non-whitelist triples. After pre-processing, the KG is used for downstream tasks by predicting links using KG embedding methods. The performance under different experimental conditions is evaluated

We study the relationship between knowledge graph relation quality and network topology by conducting pre-processing perturbations of the KG before inference time and analyzing the downstream effect on performance. This framework elucidates the relative importance that the model places on relational knowledge versus relying primarily on topology. We measure this effect by evaluating the drop in performance when corrupting relations under different network topologies. We provide a schematic illustrating the graph perturbation pipeline and subsequent evaluation for a downstream pharmacological task in Fig. 1.

### Data

We define a knowledge graph, $\mathcal {T}$, as a collection of triples of the form, $(h, r, t) \in \mathcal {T} \subseteq \mathcal {E} \times \mathcal {R} \times \mathcal {E}$, where $h, t \in \mathcal {E}$ are entities (or equivalently, nodes) and $r \in \mathcal {R}$ are relations (or equivalently, edges). Two knowledge graphs were used in this study: GNBR [4], which is NLP-derived, and Hetionet [25], which is derived from structured databases.

**Table 1:** Knowledge graph statistics. Med. ND = median node degree. Max ND = maximum node degree. EE = entity entropy (see “Metrics” section)

Dataset	$|\mathcal {T}|$	$|\mathcal {E}|$	$|\mathcal {R}|$	Med. ND	Max ND	EE
GNBR	321K	44K	32	3	8.2K	8.95
Hetionet	555K	20K	11	17	8.8K	8.89

#### GNBR

The Global Network of Biomedical Relations (GNBR) is a knowledge graph of relationships between drugs, genes, proteins, and diseases extracted from PubMed abstracts [4]. Sentences containing co-occurring pairs of drugs, genes, proteins, and diseases identified via named entity recognition (NER) were clustered together based on dependency parsing and co-occurrence frequency. Common dependency paths were assigned one of 32 high-level semantic themes by annotators, which defined 32 relations.

#### Hetionet

Hetionet is a biomedical knowledge graph comprised of structured databases from 29 sources [25]. The full KG contains 11 types of nodes and 24 types of edges describing interactions between genes, compounds, diseases, side effects, symptoms, pathways, and other entity types. We restricted the graph to chemicals, genes, proteins, and diseases to enforce comparable mechanistic-based knowledge to drive repurposing inference. Data for GNBR and Hetionet were downloaded from the compiled Drug Repurposing Knowledge Graph (DRKG) network [26]. In both KGs, genes and proteins are treated as a single entity type and not disambiguated as is standard in the field. Network statistics for GNBR and the Hetionet subset used in this work are described in Table 1.

### KG pre-processing perturbations

We evaluated the effect of four knowledge graph perturbation strategies, two changing the topology of the graph and two ablating the semantics of KG relations.

#### Topology perturbation via degree-based downsampling

Knowledge graph topology was perturbed by downsampling entities or triples based on degree before embedding and evaluation. We define the degree of a node in the knowledge graph as the sum of the in- and out-edges adjacent to the node:

$$\begin{aligned} deg(i) := |\{(h, r, t) \ | \ h=i \vee t=i\}| \end{aligned}$$

In the entity downsampling condition, a fraction, $f_{hubs}$, of entities with degree above the $p^{th}$ percentile, $deg_p$, were removed uniformly at random.

In triple-based downsampling, triples were removed from the graph based on degree until a fraction of the initial triples, *d* remained. We define the degree of a triple, *e*, as the sum of the degrees of its two entities:

$$\begin{aligned} deg(e) = deg((h,r, t)) := deg(h) + deg(t). \end{aligned}$$

To account for the correlation of triples’ degrees, whereby removal of one triple might effect the degree of another, downsampling was done iteratively in batches using Algorithm 1 where

**Figure:**
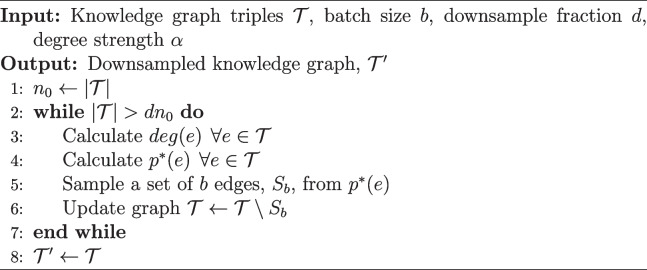
**Algorithm 1** Degree-based KG triple downsampling protocol

$$\begin{aligned} u^{*}(e) := (1+deg(e))^{\alpha } \end{aligned}$$

and

$$\begin{aligned} p^{*}(e) := u^{*}(e) / \sum \limits _{e \in \mathcal {T}} u^{*}(e). \end{aligned}$$

The degree strength parameter, $\alpha$, informs how degree is used for downsampling, as the magnitude of $\alpha$ controls the strength of the degree-based selection and the sign controls whether high-degree triples (positive $\alpha$ values) or low-degree triples (negative $\alpha$ values) have greater probability mass for downsampling.

#### Relation perturbation experiments

Two pre-processing procedures were employed to ablate biologically meaningful semantics of triples in the input knowledge graphs: flattening and corrupting. In the corrupting condition, a fraction of non-whitelist triples, $f_{corrupt}$, were corrupted, where corrupting is defined as resampling the triple’s relation to another relation, $r' \in \mathcal {R}$, uniformly at random. The flattening procedure is analogous: the relations of a fraction, $f_{flat}$, of non-whitelist triples, were mapped to a single arbitrary relation, “relates”.

### Models

#### Knowledge inference models

In this study we considered four knowledge graph embedding models for knowledge inference, TransE [27], DistMult [28], ComplEx [29], and RotatE [30]. These models map concepts and relations to discrete numerical embeddings in vector space such that knowledge graph triples have a meaningful geometric interpretation in the learned space. This representation enables downstream tasks including knowledge inference by measuring the plausibility of inferred triples, those not seen in training. In this work, embeddings are used for our knowledge reconstruction task where we infer known but obscured whitelist relationships.

In TransE, entities and relations are mapped to *k*-dimensional vectors vectors such that triples, (*h*, *r*, *t*), in the KG can be represented as translations from ${\textbf {h}}$ to ${\textbf {t}}$ via ${\textbf {r}}$, where ${\textbf {h}}, {\textbf {r}}, {\textbf {t}} \in \mathbb {R}^k$. The TransE score function is:

$$\begin{aligned} f(h, r, t) = -||{\textbf {h}} + {\textbf {r}} - {\textbf {t}}||_2 \end{aligned}$$

The notion of learning embeddings to optimize for translation is conceptually simple but fails to capture properties that may be intrinsically semantically important like symmetry.

DistMult [28] learns embeddings using a semantic matching approach, optimizing for embeddings of head, relation, and tail nodes in KG triples to point in the same direction in the real plane. The scoring function for DistMult is:

$$\begin{aligned} f(h, r, t) = {\textbf {h}}^T diag({\textbf {r}}) {\textbf {t}}, \end{aligned}$$

where $${\textbf {h}}, {\textbf {r}}, {\textbf {t}} \in \mathbb {R}^k.$$

ComplEx [29] uses a semantic approach like DistMult, but in the complex plane, ${\textbf {h}}, {\textbf {r}}, {\textbf {t}} \in \mathbb {C}^k$:

$$\begin{aligned} f(h, r, t) = Re({\textbf {h}}^T diag({\textbf {r}}) {\textbf {t}}). \end{aligned}$$

Finally, RotatE [30] learns embeddings such that the relation embedding represents a rotation of the head vector to the tail vector in the complex plane, ${\textbf {h}}, {\textbf {r}}, {\textbf {t}} \in \mathbb {C}^k$:

$$\begin{aligned} f(h, r, t) = ||{\textbf {h}} \circ {\textbf {r}} - {\textbf {t}}||^2_2, \end{aligned}$$

where $\circ$ denotes the Hadamard product. This model has been shown to be the most expressive of the four methods with the ability to capture symmetric, antisymmetric, inversion, and composition properties in relations. We focused our investigation on TransE, the model with the simplest geometric interpretation, and RotatE, the model that is the most expressive and consistently outperforms the other three on KG prediction tasks [30].

#### Implementation details

Model training and evaluation was done using the PyKEEN package [31]. Hyperparameter values were set based on existing work on hyperparameter tuning of KG embeddings for biomedical link prediction, particularly for Hetionet [17]. Models were trained for 500 epochs with learning rate = 0.02, and 50 negative samples generated per positive. The PyKEEN default embedding dimensions were used: $k = 50$ for TransE and DistMult, $k = 200$ for ComplEx and RotatE. In all experiments, we used the negative sampling loss with self-adversarial training [30] with AdaGrad [32] for optimization.

**Table 2:** Task-specific whitelist relations

Dataset	Drug-Disease	Drug-Gene	Disease-Gene
**GNBR**	Treats (T)	Binds (B)	Causal Mutations (U), Role in Pathogenesis (J), Mutations Affect Disease Course (Ud), Polymorphisms Alter Risk (Y), Promotes Progression (G)
**Hetionet**	Treats (CtD)	Binds (CbG)	Associates (DaG)

### Evaluation

Performance was evaluated on a held-out test set using a typical KG embedding evaluation framework based on concealing and inferring head and tail nodes in test triples [31].

#### Pharmacological evaluation tasks

We evaluate three different biomedical knowledge inference tasks: drug-disease prediction (drug repurposing), disease-gene association, and drug-target (equivalently “drug-gene”) interaction. For each task, a set of relations from each dataset are considered whitelist relations which are candidates for test set sampling. Whitelist relations are listed in Table 2. These comprise the standard set of whitelist relations for various pharmacological knowledge inference tasks [15].

#### Test set sampling

To split triples into training and test sets, candidate test triples were first determined after all network pre-processing. For a given task, a triple, (*h*, *r*, *t*), is eligible for inclusion in the test set if it satisfies two criteria: a) the triple’s relation, *r*, is in the whitelist set of relations for the task, and b) $min(deg(h), deg(t)) \ge 4$. We sampled 5% of permissible triples to create a test set. All other triples, including those consisting of a whitelist relation, comprised the training set.

#### Metrics

As in [23], we calculated entity entropy (EE) as a global metric of network topology. The intuition for this metric is that hubbier networks will have lower EE and networks where each node has approximately the same degree will have high EE. EE is calculated as:

$$\begin{aligned} EE(\mathcal {T}) = \sum \limits _{n \in \mathcal {E}} -P_{ESP}(n) \log P_{ESP}(n), \end{aligned}$$

where $P_{ESP}$ is the entity selection probability distribution, which describes the probability that an entity appears in a triple sampled uniformly from $\mathcal {T}$. $P_{ESP}$ is calculated as:

$$\begin{aligned} P_{ESP}(n) := \frac{|\{(h, r, t) \ | \ h=n \vee t=n\}|}{2|\mathcal {T}|}. \end{aligned}$$

Lastly, we define normalized entity entropy, $EE_{norm}$, as $EE_{norm}(\mathcal {T}) := \frac{EE(\mathcal {T})}{\log (|\mathcal {E}|)}$ such that $EE_{norm}: \mathcal {E} \times \mathcal {R} \times \mathcal {E} \rightarrow [0,1]$.

Knowledge inference performance was evaluated using adjusted mean rank index (AMRI) scores as in [16]. AMRI is a metric that considers the expectation of where entities would rank under a random uniform distribution, which is a more faithful representation of the quality of embedding-based predictions. AMRI scores lie in the range $[-1, 1]$ where a value of 1 indicates perfect performance (i.e. the obscured entity is always ranked at the top of the predicted list) and 0 indicates random-like predictions.

**Fig. 2:**
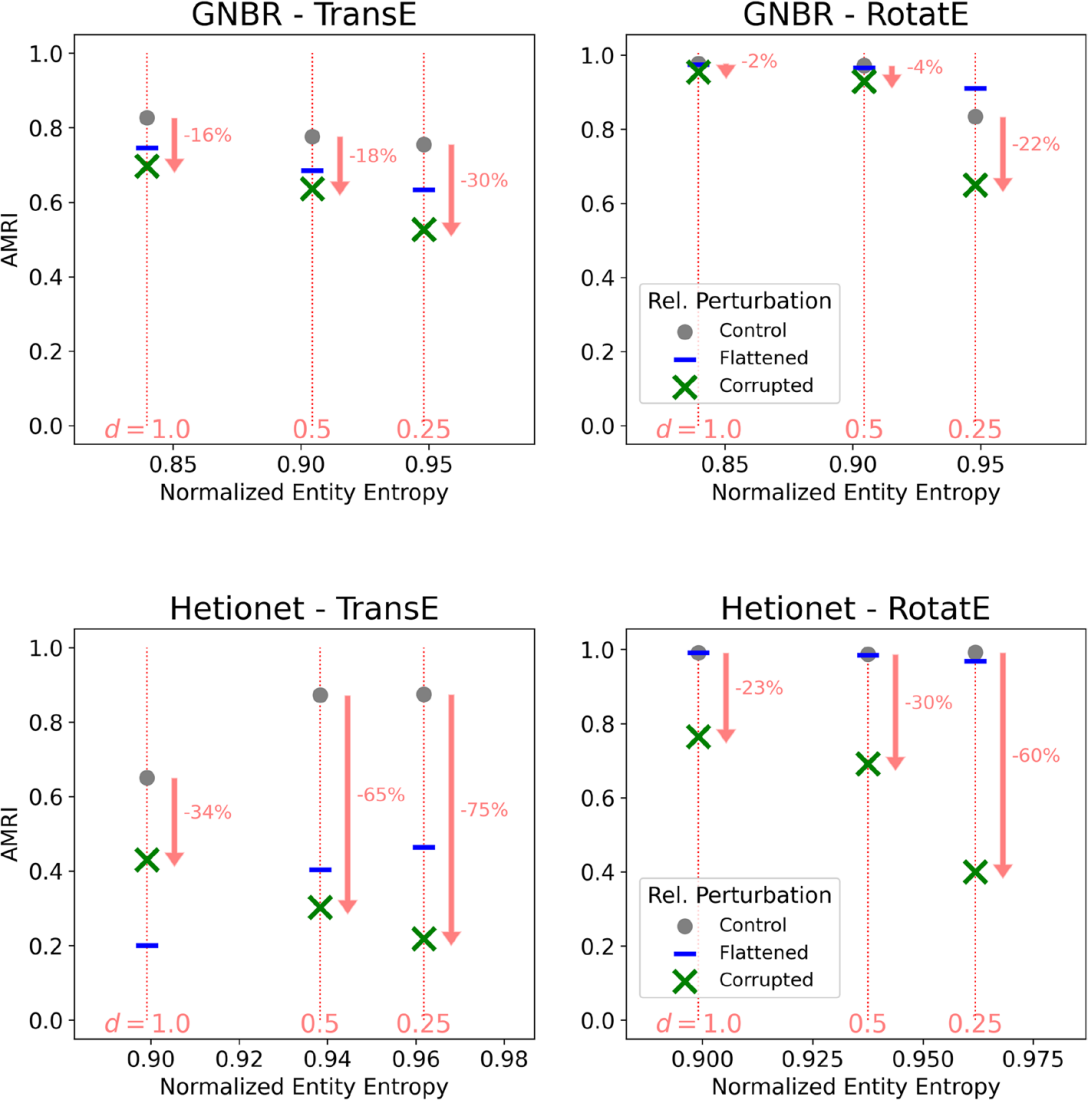
AMRI evaluation of drug-disease inference for two knowledge graphs (GNBR and Hetionet) and two KG embedding inference methods (TransE and RotatE). Results are reported for varying entity entropy conditions induced by downsampling high-degree triples ($\alpha = 2)$. Relation semantics were perturbed by two procedures: corrupting, or shuffling relations randomly, and flattening, or mapping all non-whitelist relations to a single arbitrary relation

## Results

### Main results

Drug-disease prediction for GNBR performance dropped by 16% and 2% under relation corruption for the input KG ($d = 1.0$) using TransE and RotatE. The performance drop increased to 30% and 22% upon downsampling to a quarter of triples ($d = 0.25$), favoring high-degree triples for downsampling ($\alpha = 2$). The pattern recurred for Hetionet, with performance dropping by 34% and 23% under relation corruption for TransE and RotatE without downsampling the KG, and by 75% and 60% downsampling to $d = 0.25$. In both cases, decreasing values of *d* led to more dramatic drops in performance from corrupting relations. Decreasing values of *d* did not drastically or consistently affect the performance of the KG without relation perturbation (control condition). Drops in performance of the model under relation flattening were largely insensitive to the varying levels of downsampling (Fig. 2).

### Sensitivity to degree-based downsampling

The $\alpha$ parameter was altered to preferentially downsample low-degree triples ($\alpha = -2$) or downsample triples uniformly at random ($\alpha = 0)$. In GNBR, at $\alpha = -2$, the corruption performance drop increases from 15% without downsampling to 8% at $d = 0.25$ for TransE and from 2% to 6% at $d = 0.25$ for RotatE. At $\alpha = 0$, the corruption drop in performance was relatively constant for TransE at different downsample levels and only increases from 2% to 7% in the case of RotatE. Note, the range of entity entropy values at corresponding levels of downsampling is smaller as would be expected under degree-agnostic downsampling (Fig. S1).

In the case of Hetionet, performance drop trends are largely agnostic of the downsampling low-degree triples or downsampling uniformly. At $\alpha = -2$, using TransE the performance drop under relation corruption is 32% without downsampling, but interestingly a large performance gain is seen at $d = 0.25$. With RotatE the performance drop decreases from 22% to 18% at $d = 0.25$. Under uniform downsampling, the corruption performance drop only increases from 30% $(d = 1)$ to 39% $(d = 0.25)$ with TransE and from 23% to 28% with RotatE, again noting that the changes in entity entropy are small (Fig. S2).

### Tasks beyond drug repurposing

We evaluated the reproducibility of the corruption-topology effect from the drug-disease task to two other tasks: drug-gene association and drug-target binding under high-degree triple downsampling ($\alpha = 2$). For drug-target binding, the performance drop increases from 16% $(d = 1)$ to 51% $(d = 0.25)$ for TransE and from 4% to 48% for RotatE in GNBR. Similarly, the performance drop increases from 34% $(d = 1)$ to 70% $(d = 0.25)$ in TransE and from 18% to 38% in RotatE (Fig. S3).

For the disease-gene association task, in GNBR, the performance drop increases from 18% $(d = 1)$ to 55% $(d = 0.25)$ for TransE and from 2% to 31% for RotatE. With Hetionet, the performance drop increases from 32% $(d = 1)$ to 71% $(d = 0.25)$ using TransE and from 7% to 38% using RotatE (Fig. S4).

### Additional knowledge inference models

We compared the effect of downsampling high-degree triples ($\alpha = 2$) for two other models, DistMult and ComplEx, as well. When using DistMult for inference, corrupting led to a 5% drop in performance for GNBR and 30% drop for Hetionet without downsampling. With downsampling at $d = 0.25$, performance dropped by 84% and 67% for GNBR and Hetionet, respectively.

For ComplEx, corrupting led to a 4% drop for GNBR and a 35% drop for Hetionet without downsampling triples. At $d = 0.5$ performance under corruption dropped by 22% and only 4% at $d = 0.25$ for GNBR, noting that performance of the uncorrupted network declines at increasing levels of sparsity. For Hetionet, at increasing levels of sparsity, the uncorrupted performance stays high, but the drop from corruption increases from 35% at $d = 1$ to 83% at $d = 0.5$ and 87% at $d = 0.25$ (Fig. S5).

### Comparison against removing hubs

We compared the procedure for increasing the entity entropy via triple downsampling against removing hub nodes, setting $f_{hub} = 1$ in all conditions. Using GNBR and Hetionet with TransE and RotatE for drug-disease prediction, the corruption performance drop was approximately the same for the input KGs without removing hubs $(p = 1)$ and with removing hubs of degree above the $99^{th}$ percentile $(p = 0.99)$. When downsampling at $p = 0.9$, corruption performance increased from 17% to 45% for GNBR using TransE and from 3% to 55% for GNBR using RotatE. For Hetionet, corruption performance increased from 38% at $p = 1$ to 59% at $p = 0.9$ using TransE and from 24% to 38% using RotatE (Fig. S6).

## Discussion

Inferring new knowledge implied from existing scientific findings is a powerful paradigm for discovery, particularly in pharmacological tasks. However, it is important to carefully consider biases present in the underlying data and models that drive inference. Models for knowledge inference have been shown to be susceptible to topology biases were model performance is driven primarily by predictions concerning hubby nodes.

In this work, we interrogated knowledge graph topology as a confounding factor for making use of relation semantics, which represent biologically meaningful interactions. We established a framework of ablating biological semantics by corrupting the relations in KG triples and investigated the effect on model performance under different network topologies. We found that the greatest drop in performance due to relation corruption arose in settings with higher entity entropy, where there are relatively fewer hubby nodes dominating model performance. This suggests that in higher entity entropy circumstances, the model must rely on the relations between entities rather than on node degree alone.

We observed that these results were consistent in a variety of settings. We primarily focused on the drug repurposing task but observed similar trends in drug-target prediction and disease-gene association. These findings were reflected in TransE and RotatE, our primary knowledge inference models of investigation, but also in ComplEx and DistMult representing a variety of geometric interpretations for knowledge graph embedding. Additionally, we observed this increase in corruption performance drop when simply removing a fraction of nodes with the greatest degree rather than downsampling high-degree triples. As controls, we saw that the magnitude of effect was much diminished when downsampling triples uniformly randomly or when preferentially downsampling low-degree triples.

In comparing embedding models for inference, we note interesting behaviors. Performance was consistently higher for RotatE than for TransE, which reflects that RotatE is more expressive as it can model the symmetry property for relations, and there are multiple symmetric biomedical relations in our graphs including “binds” and “associates”. This may also account for aberrant behavior such as why performance increases in the Hetionet control condition at increasing levels of sparsity, which results in a less constrained optimization problem. Further, the pronounced drop in performance when corrupting relations at increasing levels of sparsity for RotatE suggests the models’ improved capacity to find geometric mappings of semantics without introducing noise.

There are multiple implications of this work. First, we reaffirmed the prevailing notion that embedding based approaches for knowledge graph inference rely heavily on network topology and the effect of ablating semantics can be seen once the confounding of network topology is mitigated. Second, methods must be developed that properly consider edge semantics to enable true logic-like inference leveraging biological principles found in relations (e.g. [33]). Without this, methods are vulnerable to over-optimizing on network structure, which could represent an artifact of noise and data biases, such as which domains have received the most funding and thus the most has become known through scientific investigation. Third, the development of methods to mitigate the dominance of network topology for knowledge inference are prudent. Such methods could include pre-filtering knowledge based on relevant biological context or relation confidence, re-weighting knowledge to enable equal contributions to learning across different biological subdomains, implementing a Bernoulli sampler for generating negative triples according to node degree, and selectively prioritizing knowledge in optimization to lead to non-redundant, non-obvious discoveries [14].

This work has limitations as well. We limit the scope of our investigation to two global knowledge graphs, one derived from structured databases and one from unstructured text. As methods for relation extraction continue to improve [12, 34], our KGs will become higher fidelity representations of known biology from scientific research, thus these observations will be less affected by noise incurred in NLP pipelines. We also are only able to control entity entropy via a downsampling procedure, thus our observations also reflect a loss of knowledge affecting performance.

Future research directions will benefit from method development that prioritizes relation semantics with at least equal weight as network topology for capturing structure and driving logic-based inference for knowledge discovery. GNN methods [35] are well-suited to capture network structure in conjunction with entity and relation features to learn a more holistic picture of knowledge. Additionally, methods for semantic interpretability will help shed light on the degree to which relations impact inference and can help surface patterns of logic that inform the model (e.g. [36]). GPT-based chain-of-reasoning [37] work also presents a promising avenue of exploration for making semantic contributions to inference explicit.

## Conclusions

In this work we probed the interrelation between knowledge graph relational semantics and network topology as a confounding factor for knowledge graph inference. We created a framework for perturbing KG topology and KG semantics for two global, biomedical KGs, one derived from text via an NLP pipeline and one from structured data sources. We demonstrated that the drop in RotatE performance from corrupting relations increases from a 2% drop in GNBR and a 23% drop in Hetionet to a 22% and 60% drop, respectively, when downsampling highly connected triples. We showed that these results are agnostic to several embedding methods and multiple inference tasks yet specific to downsampling high-degree triples and not to downsampling low-degree triples or downsampling uniformly. This work motivates the need for further research into knowledge representation strategies that mitigate biases in highly hubby network topologies, optimization strategies that upweight low-degree yet important knowledge, and methods that emulate logic-based reasoning rather than relying on structure alone for driving KG inference. Code and analyses are provided as a Python package^1^.

### Supplementary Information

**Supplementary Material 1.**

## Data Availability

Code and analyses are provided as a Python package at https://github.com/dnsosa/kgemb-sens.

## Abbreviations

AMRI: Adjusted Mean Rank Index
DRKG: Drug Repurposing Knowledge Graph
EE: Entity Entropy
ESP: Entity Selection Probability
GNBR: Global Network of Biomedical Relations
KG: Knowledge Graph
ND: Node Degree
NER: Named Entity Recognition
NLP: Natural Language Processing
PyKEEN: Python KnowlEdge EmbeddiNgs

## Footnotes

1 https://github.com/dnsosa/kgemb-sens
