# Supplementary material for: Elucidating the semantics-topology trade-off for knowledge inference-based pharmacological discovery
Source: J Biomed Semantics. 2024 May 1;15:5. doi: 10.1186/s13326-024-00308-z (PMC11064343; doi:10.1186/s13326-024-00308-z)
Supplement: Supplementary file 1 — Supplementary Material 1. [file 13326_2024_308_MOESM1_ESM.pdf]

Supplementary Figures

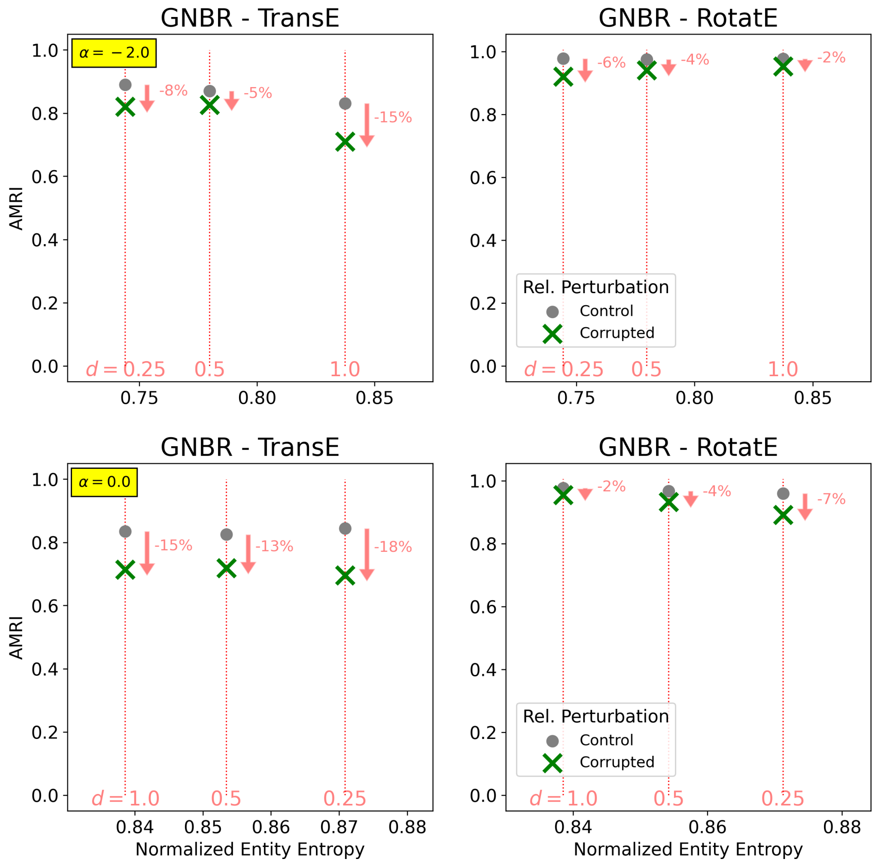

Figure S1: AMRI evaluation of drug-disease inference for GNBR using TransE and RotatE, where topology is perturbed by downsampling low-degree triples ($\alpha = -2$, top row) or downsampling triples uniformly ($\alpha = 0$, bottom row).

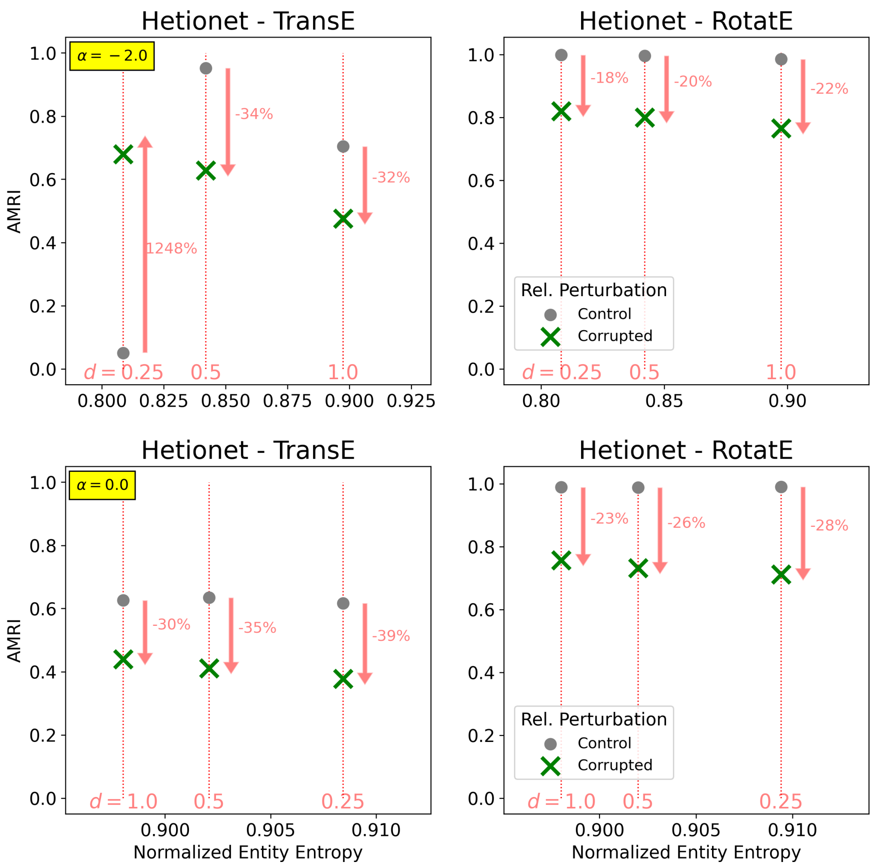

Figure S2: AMRI evaluation of drug-disease inference for Hetionet using TransE and RotatE, where topology is perturbed by downsampling low-degree triples ($\alpha = -2$, top row) or downsampling triples uniformly ($\alpha = 0$, bottom row).

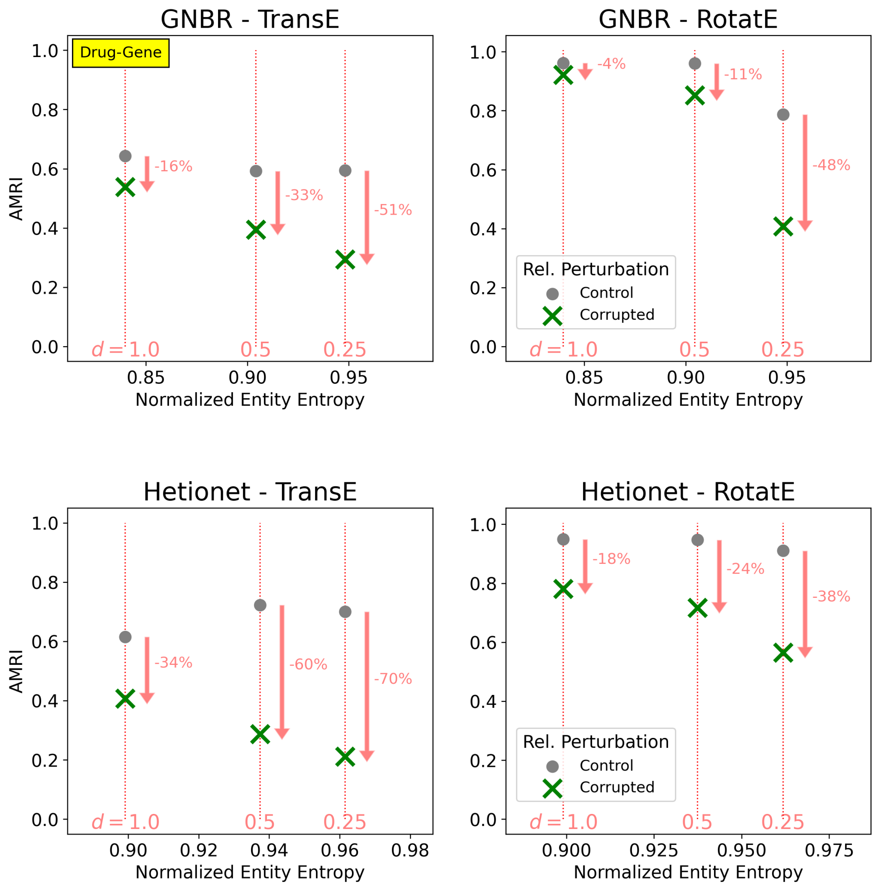

Figure S3: AMRI evaluation of drug-target predictions where topology is perturbed by high-degree triple downsampling ($\alpha = 2$).

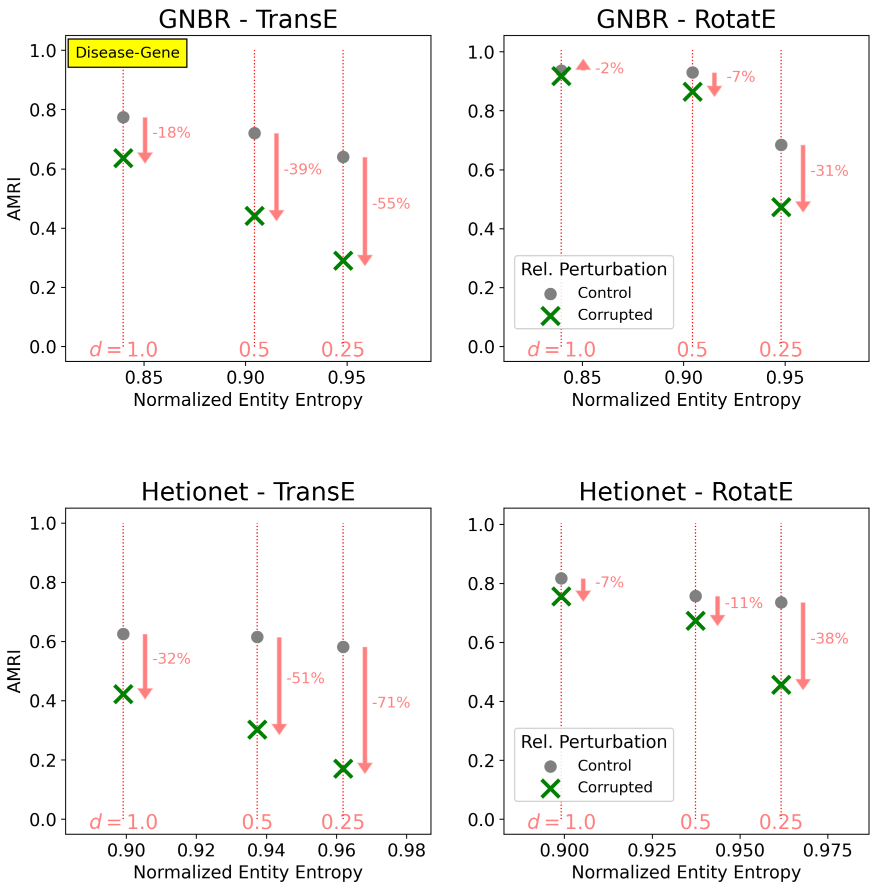

Figure S4: AMRI evaluation of disease-gene predictions where topology is perturbed by high-degree triple downsampling ($\alpha = 2$).

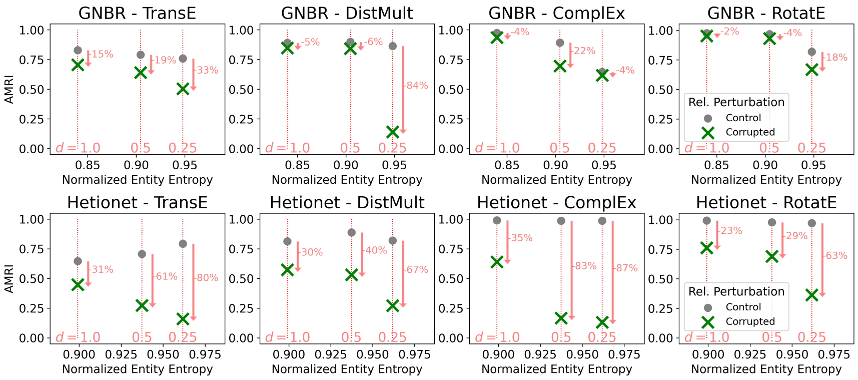

Figure S5: AMRI evaluation of drug-disease predictions where topology is perturbed by high-degree triple downsampling ($\alpha = 2$) comparing four models for knowledge graph embedding: TransE, DistMult, ComplEx, and RotatE.

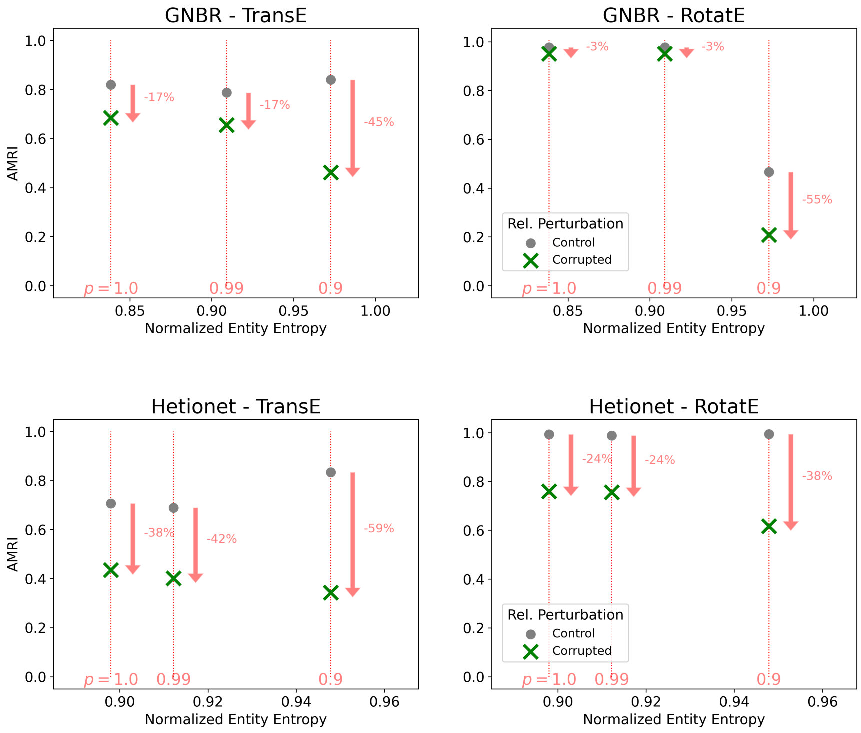

Figure S6: AMRI evaluation of drug-disease predictions where topology is perturbed by removing hubs above various degree thresholds, $p \in \{1, 0.99, 0.9\}$.
